# Knowledge structure in nursing research on infection control: application of topic modeling

**DOI:** 10.3389/fpubh.2026.1781789

**Published:** 2026-04-10

**Authors:** Dajung Ryu, Minkyung Gu, Hyun Baek, Sohyune Sok

**Affiliations:** 1 Department of Nursing, Kyungmin University, Uijeongbu-si, Gyeonggi-do, Republic of Korea; 2 Department of Nursing, College of Health Science, Daejin University, Pocheon-si, Gyeonggi-do, Republic of Korea; 3 Department of Nursing, Graduate School, Kyung Hee University, Seoul, Republic of Korea; 4 College of Nursing Science, Kyung Hee University, Seoul, Republic of Korea

**Keywords:** hygiene, infection control, knowledge, nursing research, topic modeling

## Abstract

**Background:**

Given the increasing emphasis on infection control, a comprehensive analysis of nursing research is essential for mapping prevalent scholarly themes, thereby identifying the thematic priorities and evolving knowledge structures that underpin optimized infection control strategies.

**Objective:**

This study aimed to identify meaningful concepts and trends in infection control and expand the scope of research with new insights.

**Methods:**

This exploratory study utilized Latent Dirichlet Allocation topic modeling to identify research trends over time. A total of 2,651 papers published between 1974 and 2022 were retrieved from nine databases, including PubMed, CINAHL, and Scopus.

**Results:**

Topic modeling identified four core themes, led by epidemiological investigation at 33.82% and infection control leadership at 24.06%, both of which emerged as hot topics with significantly increasing trends. Hygiene management of vulnerable subjects at 22.06% and transmission route blocking at 20.06% were identified as Cold topics, showing a significant decline in research focus over the study period.

**Conclusions:**

This study elucidates the multidimensional knowledge structure of infection control research in nursing at both micro and macro levels. The findings suggest that enhancing infection control implementation may be supported by addressing organizational factors, including leadership and professional supervision. Furthermore, these results underscore the potential for integrating epidemiological evidence into multifaceted nursing practices to strengthen infection control systems.

## Introduction

Healthcare-associated infections (HAIs) remain a major global public health challenge, imposing a substantial burden on healthcare systems worldwide. The COVID-19 pandemic has further underscored the vulnerability of healthcare settings to infectious threats and the critical importance of robust infection prevention and control strategies (1). HAIs negatively impact the quality of care, the economy, ethics, and society (2, 3), prompting the World Health Organization (WHO) to establish seven strategic goals for infection control to improve patient safety by 2030 (4).

Infection control refers to practical measures that prevent and control the harm and spread of HAIs in healthcare institutions and communities (5). Research in this field has evolved through the hierarchical stages of nursing theory proposed by Dickoff and James (6), including factor-isolating (7), factor-relating (8), situation-relating (9), and situation-producing (10) theories. While these frameworks provide a conceptual foundation for nursing knowledge, contemporary evidence has largely been synthesized through methodological approaches such as systematic reviews and meta-analyses (11, 12). Existing studies primarily focus on specific problems, interventions, comparisons, and outcomes, facilitating structured evidence synthesis.

Although systematic reviews and meta-analyses synthesize findings within predefined research questions, and bibliometric analyses map citation patterns and publication metrics, these approaches do not reveal the latent semantic structure embedded in large textual corpora (13). Consequently, they offer limited insight into how research themes co-occur, interact, and evolve over time across the broader literature (13, 14). To advance infection control research, it is necessary not only to synthesize findings but also to understand how research themes are structured and interconnected across time. Such structural insight supports more coherent knowledge development and practical application.

Topic modeling addresses this limitation by identifying hidden thematic clusters based on word co-occurrence patterns, enabling a data-driven exploration of how research themes emerge and dynamically develop without relying on predefined categories (14, 15). By uncovering the semantic organization of extensive textual data, topic modeling provides a complementary perspective for understanding the evolving knowledge structure of infection control research (16).

The emphasis on infection control underscores the need for comprehensively analyzing accumulated research to identify meaningful concepts and expand the scope of nursing insights. By utilizing topic modeling to uncover hidden patterns, this study systematically explores the knowledge structure of this field. Particularly, this study aims to (a) identify and characterize the main topics within this domain; (b) confirm the importance and trends of each theme over time; and (c) generate a schematic overview of the overall research landscape.

## Method

### Study design

This study is an exploratory design employing text mining and Latent Dirichlet Allocation (LDA) topic modeling analysis of bibliographic abstracts.

### Study subjects and data collection

The data include texts of selected nursing research abstracts about infection control, with the most recent studies published in December 2022. The search and collection of data were conducted between April and June 2023. International data were collected from CINAHL, Cochrane, Embase, PubMed, and Scopus while local data were gathered from the National Digital Science Library (NDSL), Research Information Sharing Service (RISS), Korean studies Information Service System (KISS), and Korean Citation Index (KCI). The study excluded non-journal articles (e.g., books, conference proceedings, letters, and review protocols), articles without abstracts, or articles in non-English. To ensure algorithmic consistency and semantic accuracy in the text mining process, the analysis was conducted using the English abstracts of the selected studies from both international and domestic databases. Nursing research on infection control was searched from 1947 publications for international sources and 1971 for local references. However, papers published from 1974 overseas and from 1996 domestically with searchable abstracts were included in the study.

The gathered data comprised MeSH (Medical Subject Headings) terminology and natural language. The naming of infection control in MeSH terminology was registered in 1966, and the terminology was classified as of the year 1992. Focusing on “infection control,” the previous indexes, “communicable disease control”, “cross infection prevention and control,” “infection prevention and control”, “infection management”, and “nosocomial infection control” were included. “nurs^*^” was added to limit the search strategy to nursing research associated with infection control (Appendix 1).

Of the 4,008 papers searched in international databases, 2,431 papers were selected, excluding 642 duplicate papers, 884 papers that were not associated with infection control, 31 irrelevant types of papers, and 20 papers without abstracts. Of the 612 papers searched in the national database, 220 papers were selected, excluding 232 duplicate papers, 145 papers that were not linked to infection control, 7 irrelevant types of papers, and 8 papers without abstracts. The final 2,651 papers were used for analysis (Appendix 2).

## Data analysis

### Extraction and refining of words

The bibliographic information, including the title, abstract, and publication year of the collected papers was exported to Endnote X8, a bibliographic management program. To extract words from the abstract, nouns were extracted using the Netminer 4.5.0 program (Cyram Inc., Seongnam). Netminer's natural language processing automatically excludes stop words such as adverbs and numbers. However, singular and plural, spacing, abbreviations, and uppercase and lowercase letters were classified into different words and need to be refined. Word refinement was conducted in three stages (a) defining compound nouns (e.g., contact precautions); (b) excluding structural indices of abstracts (e.g., methods) and ambiguous terms (e.g., solution) that lack thematic specificity; and (c) unifying synonyms and linguistic variants (e.g., nurse, registered nurse) into representative terms through lemmatization.

Subsequently, the words were refined using frequency analysis and TF-IDF (Term Frequency Inverse Document Frequency) analysis to extract frequently appearing influential words. TF is the number of times a particular word appears in a particular document. IDF is the reciprocal of how often a particular word appears in the entire document. Words with a higher reciprocal value indicate that they appear more frequently in the current document than in the entire document. It is also possible to identify specific words in a particular document and general words in the entire document. This study selected words with more than two occurrences in the document using frequency analysis. Words with a TF-IDF value of 0.7 or higher were extracted by referring to previous studies (17).

### Topic modeling

LDA algorithm was used for topic modeling to determine the number of topics and the hyperparameter α and β values set by a researcher. This study employed the silhouette index as a quantitative measure to evaluate the structural distinctiveness and clustering quality of the topics within the NetMiner environment (18). The silhouette index indicates the clustering of a topic. It has a value of −1 to 1, and the closer it is to 1, the better the clustering. While previous studies have suggested various hyperparameter ranges—typically α = 50/number of topics and β between 0.01 to 0.5 (19)—the final model for this study was constructed with 4 topics, α = 0.1, and β = 0.01, yielding a silhouette index of 0.926 (Appendix 3).

### Importance and trend by topic

The period was categorized by time and event to identify changes in the importance of topics in the total article. Papers with collected data were divided into 10-year periods, with years 1974 to 1979 as the first period, years 1980 to 1989 as the second period, years 1990 to 1999 as the third period, years 2000 to 2009 as the fourth period, years 2010 to 2019 as the fifth period, and years 2020 to 2022 as the sixth period. The category for each event was based on the onset of the pandemic (20), and the acquired immunodeficiency syndrome (AIDS) (1981), severe acute respiratory syndrome (SARS) (2002), Swine influenza (2009), Ebola (2014), and coronavirus (COVID-19) (2019) were the baseline points, with years 1974 to 1980 as the first period, years 1981 to 2001 as the second period, years 2002 to 2008 as the third period, years 2009 to 2013 as the fourth period, years 2014 to 2018 as the fifth period, and years 2019 to 2022 as the sixth period.

Linear regression analysis was conducted using SPSS 25.0 (IBM Corp., NY) to identify the trend of increased or decreased numbers of topics over time. The positive regression coefficient indicates a topic with increased interest (hot topic) in nursing research on infection control, whereas the negative one implies a topic with decreased interest (cold topic) in nursing research on infection control.

### Ethical considerations

This study uses information available to the general public, and was exempted from review by the Kyung Hee University Institutional Review Board (IRB No. KHSIRB-23-143(EA), Approval date February 12, 2023).

## Results

### Key words by topic

**Figure 1:**
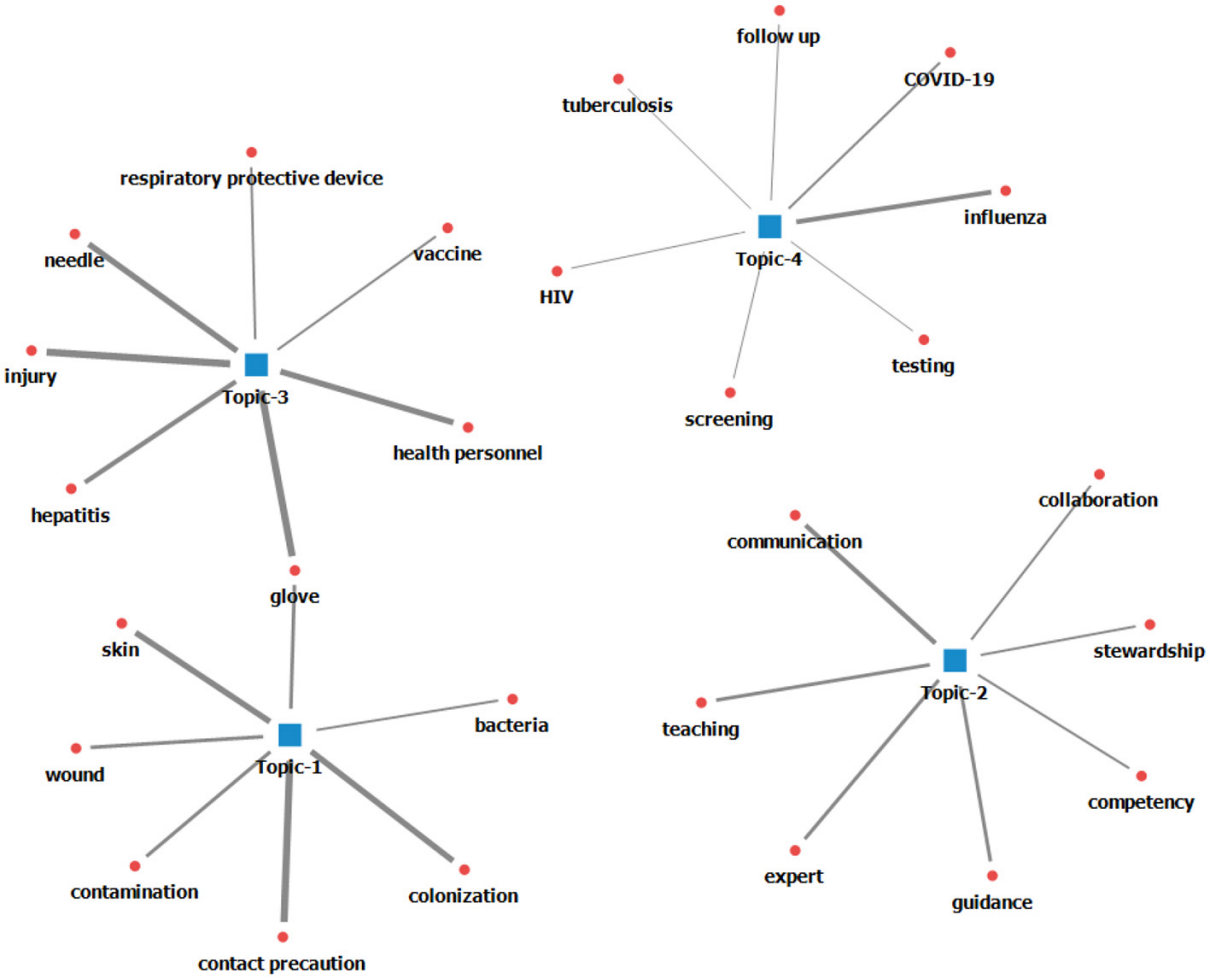
Topic specific keywords map.

The silhouette index as a quantitative method was used to determine the number of topics. When the number of topics was 4, hyperparameter α = 0.1, and β = 0.01, it was derived as 0.926, which was the closest to 1. Therefore, the topic was modeled using this setting. The keywords for each of the four topics clustered when the number of topics is 4, α = 0.1, β = 0.01, and Iteration = 1,000 times were organized in order of probability (21) (Table 1 and Figure 1).

**Table 1:** Main keywords by topic.

Classification (proportion)		Topic 1 (22.06%)	Topic 2 (24.06%)	Topic 3 (20.06%)	Topic 4 (33.82%)
Keywords (probability)	1^st^	Contact precaution	Communication	Glove	Influenza
		(0.043)	(0.030)	(0.045)	(0.032)
	2^nd^	Skin	Expert	Injury	COVID-19
		(0.036)	(0.026)	(0.044)	(0.028)
	3^rd^	Colonization	Teaching	Health personnel	Tuberculosis
		(0.036)	(0.025)	(0.036)	(0.026)
	4^th^	Wound	Guidance	Needle	Screening
		(0.028)	(0.025)	(0.036)	(0.025)
	5^th^	Contamination	Competency	Hepatitis	Follow up
		(0.025)	(0.022)	(0.028)	(0.023)
	6^th^	Glove	Collaboration	Respiratory protective device	Testing
		(0.025)	(0.019)	(0.022)	(0.021)
	7^th^	Bacteria	Stewardship	Vaccine	HIV
		(0.020)	(0.019)	(0.019)	(0.021)

### Main themes of the topics

The main themes of the topics named through the keywords are as follows: Topic 1 (22.06%) was titled “hygiene management of vulnerable subjects.” This topic covered studies concentrated on the following: First, the skin condition of the subjects or carriers was observed. Second, disinfection was conducted to reduce bacterial colonization and contamination. Third, hygiene was maintained by wearing gloves during wound care that required contact attention. Then, Topic 2 (24.06%) was named “infection control leadership.” Studies that improved the infection control capabilities of healthcare providers were categorized as follows: First, infection control experts monitored the use of antibiotics through smooth communication and collaboration between healthcare providers to prevent multidrug-resistant bacterial infections. Second, infection control experts shared their knowledge of infection control through training and imparting advice on infection control techniques. Topic 3 (20.06%) was labeled “transmission route blocking.” It encompassed studies about blocking the transmission route and discussed the following: First, healthcare providers wear gloves to minimize cross-infection caused by contact infections, such as hepatitis and needlestick injuries. Second, healthcare providers take vaccinations and wear respiratory protective equipment to block the transmission route of droplet and airborne infections. Lastly, Topic 4 (33.82%) was called “epidemiological investigation.” This topic comprised topics in the following areas: First, the studies traced the pathogens and spread of pandemic infectious diseases, such as human immunodeficiency virus (HIV), influenza, tuberculosis, and COVID-19. Second, the studies presented epidemiological characteristics of infectious diseases by monitoring infection rates through testing and screening (Table 2).

**Table 2:** Topic group.

Classification (proportion)	Topic group
Topic 1 (22.06%)	Hygiene management of vulnerable subjects
Topic 2 (24.06%)	Infection control leadership
Topic 3 (20.06%)	Transmission route blocking
Topic 4 (33.82%)	Epidemiological investigation

### Schematic overview of infection control

**Figure 2:**
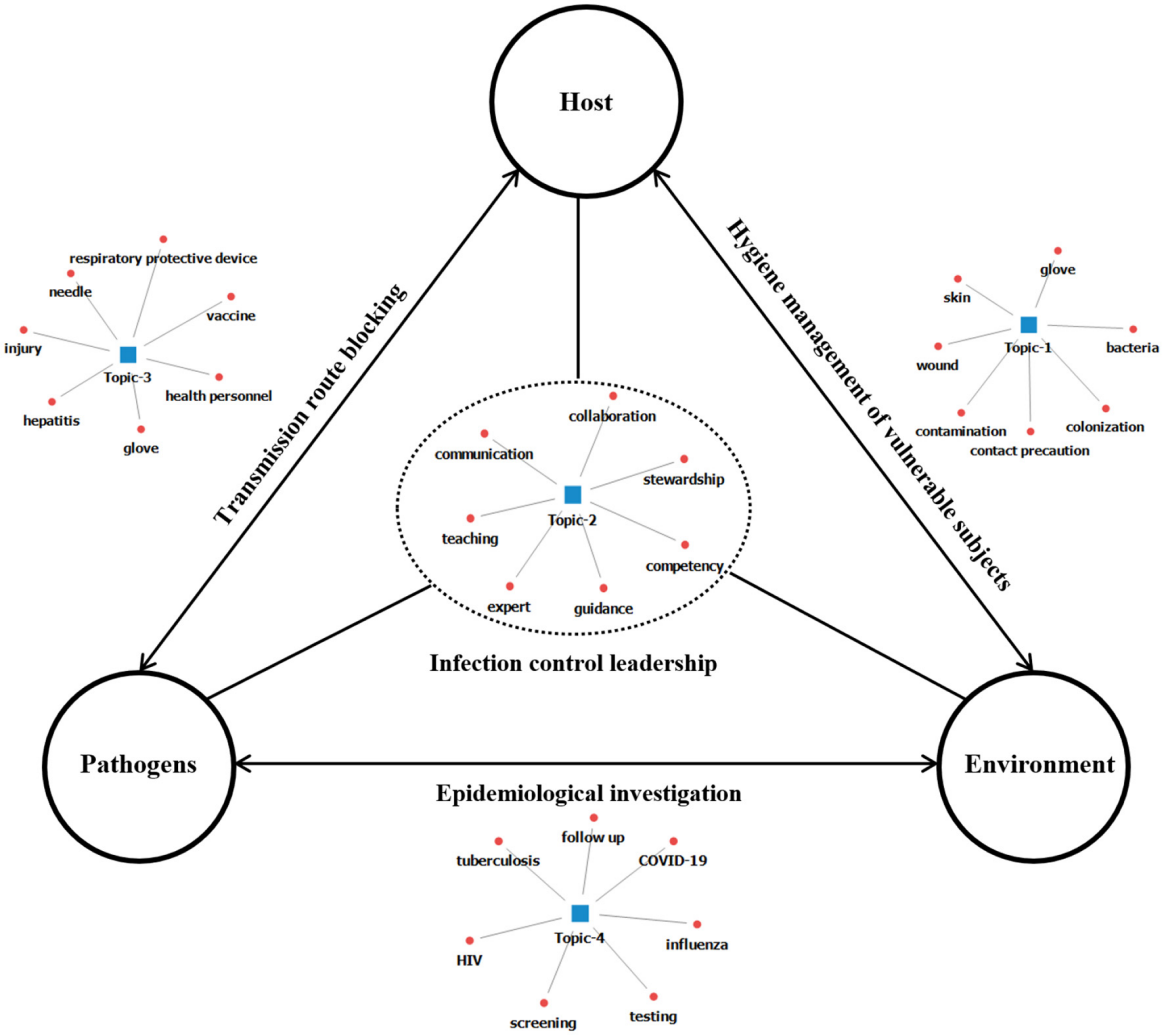
Schematic overview of infection control.

HAIs are caused by the interaction between the pathogen, the host, and the environment. This means that addressing HAI transmission should also consider the pathogen, the host, and the mode of transmission. In line with this, a schematic overview of infection control through the results of this study is presented as follows (Figure 2).

### Importance and trend by topic

**Figure 3:**
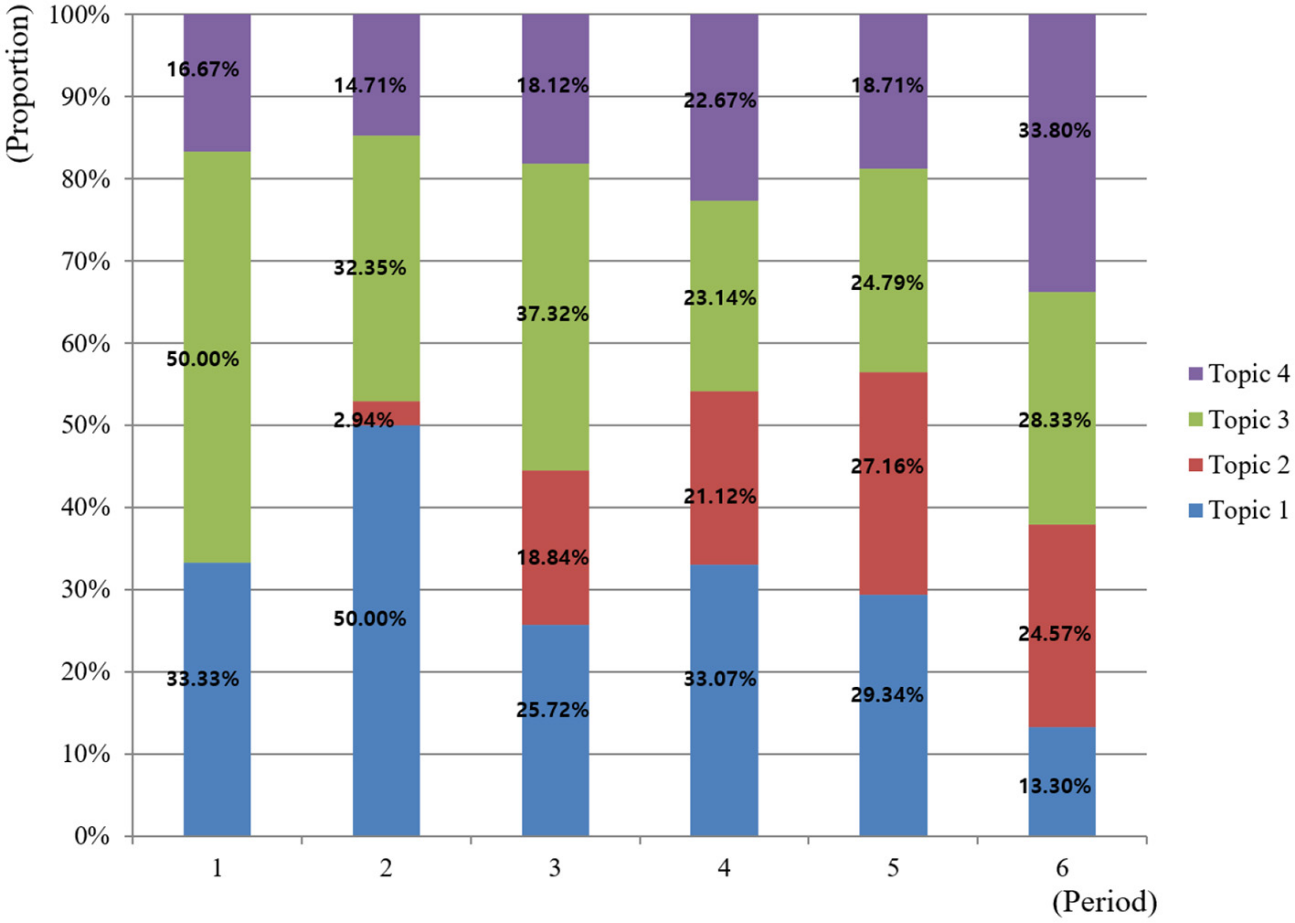
Topic proportion by period.

**Figure 4:**
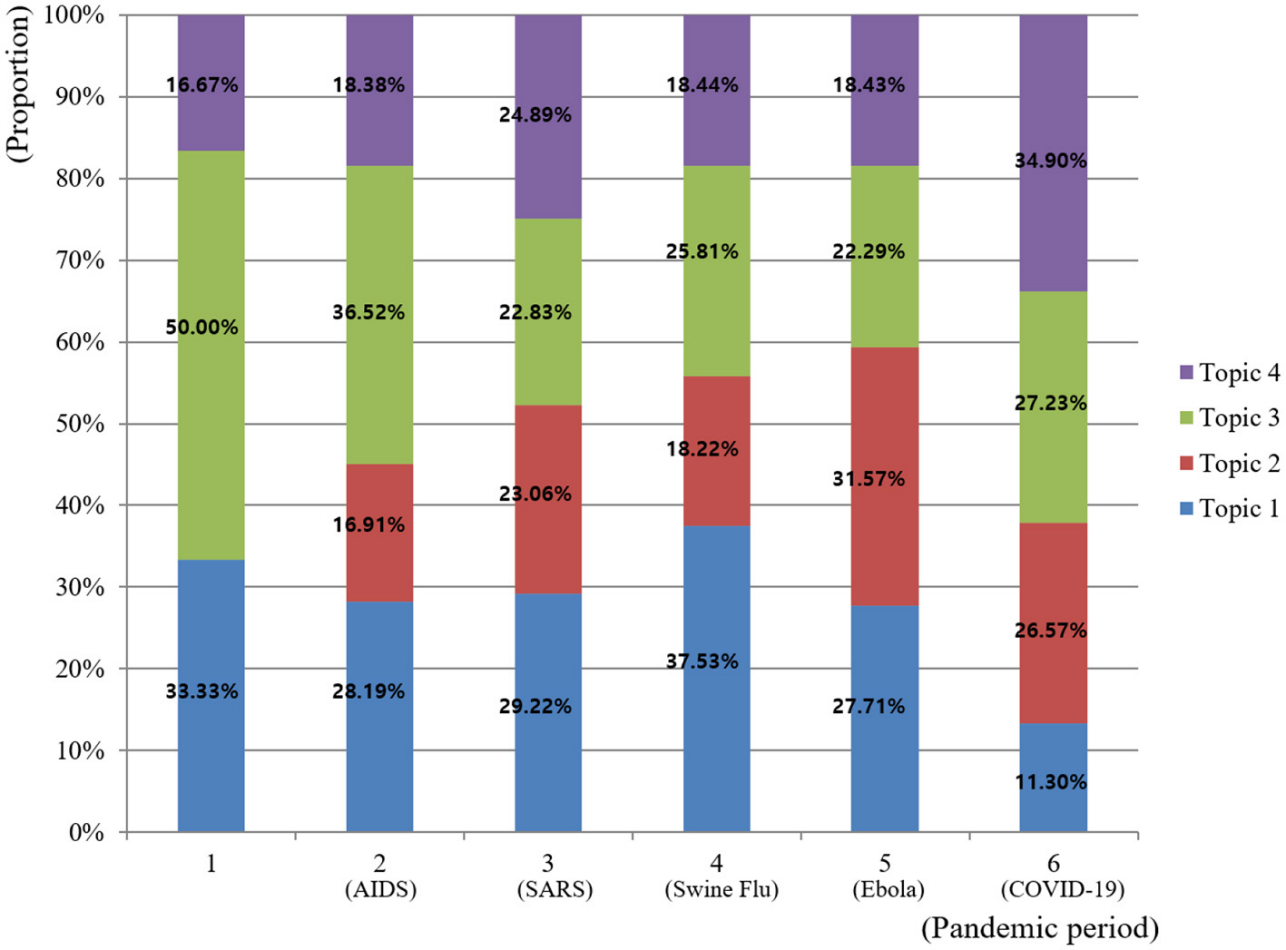
Topic proportion by pandemic occurrence period.

The changes in the importance of each topic classified by time and event are as follows (Figures 3, 4). The results of the linear regression analysis confirmed the temporal trends of the four topics (Table 3). To statistically verify the temporal trends of the research topics, we conducted individual linear regression analyses for each of the four identified topics. The dependent variable was defined as the relative proportion of the specific topic within each period, and the independent variable was chronological time period. Specifically, Topic 2 (Infection control leadership) and Topic 4 (Epidemiological investigation) showed significant positive trends (B = 0.581, *p* < 0.001; B = 0.280, *p* = 0.002, respectively), thus being classified as “Hot topics.” Conversely, Topic 1 (Hygiene management of vulnerable subjects) and Topic 3 (Transmission route blocking) exhibited significant negative trends (B = –0.270, *p* = 0.025; B = –0.591, *p* = 0.016, respectively) and were identified as “Cold topics.”

**Table 3:** Linear regression analysis of the topics.

Topic group	B	t	*p*	Type
Hygiene management of vulnerable subjects (Topic 1)	−0.270	−1.152	0.025^*^	Cold topic
Infection control leadership (Topic 2)	0.581	4.215	< 0.001^*^	Hot topic
Transmission route blocking (Topic 3)	−0.591	−2.510	0.016^*^	Cold topic
Epidemiological investigation (Topic 4)	0.280	2.298	0.002^*^	Hot topic

^*^p < 0.05.

## Discussion

Four major topics were classified using topic modeling—through determining frequent keywords that appeared in nursing research on infection control. Subsequently, the study examined the importance of the topics by categorizing them based on the time when the nursing research on infection control was conducted and the time of the outbreak of the pandemic or the occurrence of infectious diseases. The study also confirmed that the change in the significance of the topics was similar. The signs of the regression coefficient were used to classify the topics with increased interest and decreased interest and then identify the trends in nursing research on infection control by topic.

Topic 1, centered on hygiene management for vulnerable subjects, demonstrated the highest overall importance but showed a declining temporal trend (Cold topic). Historically, hygiene protocols involving chlorhexidine-containing products have been a cornerstone of infection control, particularly for ICU patients and those with multidrug-resistant infections (22, 23). However, our analysis suggests that the focus of nursing research is shifting. While traditional hygiene remains clinically vital for preventing complications such as HAIs, the decline in research interest likely reflects a saturation of evidence (24, 25). This implies that foundational hygiene management has reached a stage of empirical maturity, leading researchers to pivot toward more complex, multi-dimensional infection control strategies.

Topic 2, emphasizing leadership and organizational safety culture, was identified as a Hot topic. This aligns with the WHO's multi-strategy recommendations for establishing a culture of safety through communication and positive reinforcement (26). Our findings highlight a growing recognition of the nurse-leader role in managing dynamic clinical environments. As healthcare providers struggle to balance educator and practitioner roles, the increasing trend in Topic 2 reflects an urgent need for theoretical frameworks that support leadership development in infection control (27). This underscores that successful infection control is no longer viewed solely as a technical task, but as an organizational outcome driven by leadership competency.

Topic 3 focused on blocking transmission routes (e.g., vaccines). The observed decrease in importance (Cold topic) suggests a paradigm shift in how nurses approach transmission prevention. Traditional aseptic techniques, once the primary focus, are increasingly being integrated with or superseded by advanced vaccination protocols and evidence-based cleaning technologies (28, 29). This trend suggests that infection control methods are evolving toward more cost-effective and resource-efficient strategies without compromising clinical outcomes.

Topic 4, related to epidemiological investigations of pandemics (HIV, Influenza, COVID-19), showed a sharp increase in interest (Hot topic). The rapid spike during the sixth period (2020–2022) must be interpreted with caution; as this period covers only 3 years, it represents a significant temporal bias driven by the global COVID-19 crisis. Nevertheless, the data reveal a critical gap in nursing research: while nurses are the primary administrators of antibiotics, Topic 4 remains dominated by medical-centered research on antibiotic surveillance (30). This suggests a pressing need for nursing-led research in antibiotic stewardship to enhance patient safety and reduce multidrug-resistant infections. To bridge this gap, future research should move beyond conceptual advocacy and focus on the development and validation of nurse-led antibiotic stewardship protocols. Specifically, empirical studies are needed to evaluate the efficacy of nursing-driven interventions in clinical areas such as the early assessment of antibiotic necessity, systematic monitoring of adverse drug reactions, and patient-centered education on medication adherence. Furthermore, investigating the integration of nursing surveillance into multidisciplinary antimicrobial teams will be essential for redefining the nurse's role as a proactive monitor of antibiotic therapy rather than a mere administrator.

### Implications for practice, policy, and research

Nursing research on infection control has spanned approximately 50 years. However, the development of nursing theories about infection control appears incomplete. To address this gap, the current study utilized the epidemiological triangle—a foundational disease-oriented theory—to generate a schematic overview of infection control. This model conceptualizes the occurrence of HAIs as a dynamic interaction among pathogens, hosts, and environments, where changes in any domain can alter the invasiveness of pathogens and create new infection patterns (31). Crucially, our schematic overview extends this traditional model by positioning nursing practice as an active mediating force that intervenes across all three domains. In this framework, nursing is not merely situated within the host or environmental sphere but functions integratively to disrupt transmission pathways, strengthen host resilience, and modify environmental risk structures. Thus, the proposed schematic overview represents a translational adaptation of a disease-centered model into a practice-oriented framework grounded in nursing roles. Future studies that validate this schematic outline will be essential to support these findings and inform the development of evidence-based health policies related to infection control.

## Limitations

This study has several limitations. First, in order to establish a search strategy in the data collection stage, infection control nursing research was searched with advice from a nursing librarian, and studies agreed upon with one nursing professor were collected. Second, MeSH terms were used to extract and refine words, and natural languages that appeared in previous studies were used as limited words. Because words are expressed in different ways by each author, there may be bias in extracting keywords from the abstract. Third, the inclusion of multiple Korean databases may have introduced regional bias. In addition, the reliance on abstract-only analysis, while efficient for identifying long-term research trends in large-scale datasets, may have limited the granularity of the findings compared with full-text analysis.

## Conclusion

In conclusion, the core themes of nursing research on infection control identified through topic modeling were hygiene management of vulnerable subjects, infection control leadership, transmission route blocking, and epidemiological investigation. The results also indicate that the importance of nursing research on infection control by topic has diminished since the 2000 s in the hygiene management of vulnerable subjects. Still, infection control leadership has increased over time since its emergence in the 1980 s. The transmission route blocking accounted for an average of 33% and the use of epidemiological investigations rapidly increased from 2020. Then, in terms of the trend by topic in nursing research on infection control, the topics associated with infection control leadership and epidemiological investigation have gained increased interest. However, there has been decreased interest in the topics associated with the hygiene management of vulnerable subjects and transmission route blocking. This study is relevant as it identified the scientific knowledge structure in nursing theories for current infection control at micro and macro levels. Also, the study provided a theoretical basis for nursing interventions for infection control. In addition, it presented the scope and level of nursing theories for future infection control, which can be used in further research.

## Data Availability

The raw data supporting the conclusions of this article will be made available by the authors, without undue reservation.
