# Supplementary material for: Knowledge structure in nursing research on infection control: application of topic modeling
Source: Front Public Health. 2026 Apr 10;14:1781789. doi: 10.3389/fpubh.2026.1781789 (PMC13106434; doi:10.3389/fpubh.2026.1781789)
Supplement: Supplementary file 1 [file Table_1.doc]

**Appendix 1 Search strategy of infection control nursing research**

| **Database** | **Search No.** | **Query** |
| --- | --- | --- |
| CINAHL | #1 | MM “infection control” |
|  | #2 | TI (“infection control”[Mesh] OR “communicable disease control”[Mesh] OR “cross infection prevention and control”[Mesh] OR “infection prevention and control”[Mesh]) |
|  | #3 | S1 OR S2 |
|  | #4 | MM “Nurses+” |
|  | #5 | TI nurs* |
|  | #6 | S4 OR S5 |
|  | #7 | S3 AND S6 |
| Cochrane | #1 | MeSH descriptor: [Infection Control] explode all trees |
|  | #2 | (“infection control” OR “communicable disease control” OR “cross infection prevention and control” OR “infection prevention and control” OR “infection management” OR “nosocomial infection control”):ti,ab |
|  | #3 | #1 OR #2 |
|  | #4 | MeSH descriptor: [Nursing] explode all trees |
|  | #5 | nurs*:ti,ab |
|  | #6 | #4 OR #5 |
|  | #7 | #3 AND #6 |
| Embase | #1 | ‘infection control’/mj/exp OR ‘infection control’ OR ‘infection control’:ti,ab OR ‘communicable disease control’/mj/exp OR ‘communicable disease control’ OR ‘communicable disease control’:ti,ab OR ‘cross infection prevention and control’/mj/exp OR ‘cross infection prevention and control’ OR ‘cross infection prevention and control’:ti,ab OR ‘infection prevention and control’/mj/exp OR ‘infection prevention and control’ OR ‘infection prevention and control’:ti,ab OR ‘infection management’ OR ‘infection management’:ti,ab OR ‘nosocomial infection control’ OR ‘nosocomial infection control’:ti,ab |
|  | #2 | ‘nurses’/mj OR nurses:ti,ab OR ‘nursing’/mj OR nursing:ti,ab |
|  | #3 | #1 AND #2 |
| PubMed | #1 | “infection control”[MeSH Terms] OR “communicable disease control”[MeSH Terms] OR “cross infection prevention and control” OR “infection prevention and control” OR “infection control”[Title/Abstract] OR “communicable disease control”[Title/Abstract] OR “cross infection prevention and control”[Title/Abstract] OR “infection prevention and control”[Title/Abstract] OR “infection management”[Title/Abstract] OR “nosocomial infection control”[Title/Abstract] |
|  | #2 | “nurses”[MeSH Terms] OR “nursing”[MeSH Terms] OR “nursing research”[MeSH Terms] OR “specialties, nursing”[MeSH Terms] OR “nursing, supervisory”[MeSH Terms] OR “nurses”[Title/Abstract] OR “nursing”[Title/Abstract] OR “nursing research”[Title/Abstract] OR “specialties nursing”[Title/Abstract] OR “nursing” |
|  | #3 | #1 AND #2 |
| Scopus | #1 | TITLE-ABS-KEY((“infection control” OR “communicable disease control” OR “cross infection prevention and control” OR “infection prevention and control” OR “Infection management” OR “nosocomial infection control”) AND nurs*) AND ( LIMIT-TO ( SUBJAREA,“NURS” ) ) AND ( LIMIT-TO ( LANGUAGE,“English” ) ) |

**Identification of studies via databases and registers**

Records identified from:

CINAHL (n = 1,201)

Cochrane (n = 309)

Embase (n = 729)

PubMed (n = 950)

Scopus (n = 819)

KCI (n = 133)

KISS (n = 272)

NDSL (n = 64)

RISS (n = 143)

Records removed *before screening*:

Duplicate records removed (n = 874)

Records marked as ineligible by

automation tools (n = 0)

Records removed for other reasons

(n = 0)

**Identification**

Records screened

(n = 3,746)

Records excluded

(n = 1,029)

**Screening**

Reports sought for retrieval

(n = 2,717)

Reports not retrieved

(n = 0)

Reports assessed for eligibility

(n = 2,717)

Reports excluded:

Not journal articles (n = 38)

No abstracts available (n = 28)

**Included**

Studies included in review

(n = 2,651)

**Appendix 2** PRISMA flow diagram

Source: Page MJ, et al. BMJ 2021;372:n71. doi: 10.1136/bmj.n71.

This work is licensed under CC BY 4.0. To view a copy of this license, visit <https://creativecommons.org/licenses/by/4.0/>

**Appendix 3 Silhouette index by topic modeling**

| **Rank** | **Number of topics** | **Hyper parameter** | | **Silhouette index** |
| --- | --- | --- | --- | --- |
|  |  | ***α*** | ***β*** |  |
| 1 | 4 | 0.1 | 0.01 | 0.926 |
| 2 | 4 | 0.2 | 0.01 | 0.921 |
| 3 | 4 | 0.1 | 0.02 | 0.920 |
| 4 | 5 | 0.1 | 0.02 | 0.918 |
| 5 | 8 | 0.1 | 0.01 | 0.916 |
| 6 | 4 | 0.2 | 0.02 | 0.915 |
| 7 | 5 | 0.2 | 0.02 | 0.915 |
| 8 | 9 | 0.1 | 0.01 | 0.910 |
| 9 | 8 | 0.2 | 0.01 | 0.904 |
| 10 | 9 | 0.1 | 0.02 | 0.902 |
